# iRoot BP Plus promotes osteo/odontogenic differentiation of bone marrow mesenchymal stem cells via MAPK pathways and autophagy

**DOI:** 10.1186/s13287-019-1345-3

**Published:** 2019-07-29

**Authors:** Jiamin Lu, Zehan Li, Xiao Wu, Yan Chen, Ming Yan, Xingyun Ge, Jinhua Yu

**Affiliations:** 1 Key Laboratory of Oral Diseases of Jiangsu Province, Institute of Stomatology, Nanjing Medical University, 136 Hanzhong Road, Nanjing, 210029 Jiangsu China; 2 Endodontic Department, School of Stomatology, Nanjing Medical University, 136 Hanzhong Road, Nanjing, 210029 Jiangsu China; 3 Nanjing Stomatological Hospital, Medical School of Nanjing University, 30 Zhongyang Road, Nanjing, 210008 Jiangsu China

**Keywords:** iRoot BP Plus, BMMSCs, Osteo/odontogenic differentiation, Autophagy, MAPK pathways

## Abstract

**Background:**

iRoot BP Plus is a novel bioceramic endodontic material. Recently, it has been considered as an alternative to MTA which is the most popular scaffold cover during regenerative endodontic therapy. This study aimed to evaluate the effects of iRoot BP Plus on the osteo/odontogenic capacity of bone marrow mesenchymal stem cells (BMMSCs), including the underlying mechanisms.

**Methods:**

BMMSCs were collected by a whole marrow method and treated with iRoot BP Plus-conditioned medium (BP-CM). The proliferation ability was evaluated by cell counting kit 8 and flow cytometry. Complete medium was used as a blank control, and 2 mg/ml MTA-conditioned medium was served as a positive control. Alkaline phosphatase (ALP) activity assay, ALP staining, western blot, real-time RT-PCR, Alizarin Red S staining, and immunofluorescence staining were performed to explore the osteo/odontogenic potential and the involvement of MAPK pathways. Besides, autophagy was investigated by western blot, immunofluorescence staining, and transmission electron microscopy.

**Results:**

0.2 mg/ml BP-CM showed higher ALP activity, enhanced matrix mineralization, and upregulated osteo/odontogenic-related makers without affecting the proliferation ability of BMMSCs. In addition, there was no significant difference between the effects of iRoot BP Plus and MTA on the osteo/odontoblastic potential of BMMSCs. Mechanistically, activation of the MAPK pathways was observed in the BP-CM-treated BMMSCs, and the effect of BP-CM on cell differentiation was weakened by inhibition of the MAPK pathways. Meanwhile, the autophagy level increased during cell-committed differentiation, and suppression of autophagy downregulated BP-CM-induced osteo/odontogenic differentiation.

## Conclusion

iRoot BP Plus promotes the osteo/odontogenic differentiation of BMMSCs via the MAPK pathways and autophagy, which are important for its application in bone and tooth tissue regeneration engineering.

## Introduction

Dental pulp exposure caused by carious or trauma in immature teeth always leads to necrosis and apical periodontitis. Meanwhile, the risk of root fracture will increase due to thin root walls and shortened root length resulting from arrested root development [1]. In these cases, regenerative endodontic therapy is the most desirable healing response because of the pulp-dentine complex regeneration and further root development after treatment. Although the protocols of regenerative endodontic therapy vary considerably, biomaterial is essential in all these treatments [2]. Nowadays, mineral trioxide aggregate (MTA), a calcium silicate-based biomaterial, is used extensively for scaffold cover during regenerative endodontic therapy. Meanwhile, our previous studies revealed that 2 mg/ml MTA-conditioned medium could boost odonto/osteogenic potential in BMMSCs [3], human periodontal ligament stem cells [4], and stem cells from apical papilla [5]. However, MTA is not very friendly to clinicians because it has several disadvantages, such as long setting time, tooth discoloration, and handling characteristics [6].

iRoot BP Plus (Innovative BioCeramix Inc., Vancouver, BC, Canada) is a laboratory-synthesized, pre-mixed, ready-to-use, injectable bioceramic-based endodontic material [7]. According to the manufacturer’s instruction, the application of iRoot BP Plus includes root-end fillings, apexification, pulpotomy, and indirect or direct pulp capping. iRoot BP Plus has similar cytotoxicity, antibacterial efficacy, and root-end sealing in comparison with MTA [8–10]. Moreover, it overcomes the drawbacks of MTA. Therefore, recently, iRoot BP Plus has been considered as an alternative to MTA.

In addition to biomaterials, stem cells are another key factor in tissue engineering [11]. During regenerative endodontic therapy, blood from periapical tissues needs to be induced to the root canal system where different types of stem cells, including bone marrow mesenchymal stem cells (BMMSCs), might help regenerating bone and dentin tissues [12]. Because of their excellent proliferation and multi-lineage differentiation ability, BMMSCs have been speculated to be promising cell sources for stem cell-based clinical treatment. For better application of BMMSCs in bone/teeth tissue regeneration, researchers identified lots of small molecules and growth factors to accelerate the osteo/odontogenic differentiation process of BMMSCs. For example, Lei et al. reported that natural dentine matrix could regulate BMMSC odontogenic differentiation [13]. Moreover, 0.5 μM bisphosphonates triggered the odontogenic ability of BMMSCs [14]. However, the relationship between iRoot BP Plus and osteo/odontogenic differentiation of BMMSCs remains elusive.

It is generally known that multiple regulatory mechanisms take part in regulating multidirectional differentiation of mesenchymal stem cells (MSCs), and MAPK pathways are included. MAPK pathways consist of a number of proteins and constitute a complex cascade. There are three classic MAPK subfamilies that have been characterized well in mammals: c-Jun N-terminal kinase (JNK), extracellular signal-regulated kinase (ERK), and p38 [15]. Lots of studies report the key role of MAPKs in osteoblast differentiation. Liu et al. verified that suppression of the JNK pathway resulted in downregulation of osteogenic markers and mineralization [16]. Besides, p-p38 and p-ERK lead RUNX2 phosphorylation, which increases the transcriptional potential of RUNX2. Similarly, cooperation between the p38 and JNK pathways promote osteogenic effects [17].

Recently, the interaction between osteo/odontogenic differentiation and autophagy, which is a significant cellular biological process, has gained lots of interest. Autophagy is defined as a natural and self-cannibalization process responsible for the degradation of cytosolic proteins and subcellular organelles in lysosomes. Nowadays, several researchers have reported that autophagy has critical functions in stem cell differentiation. Interestingly, Meng et al. verified that the impaired osteogenic capacity of BMMSCs in osteoporosis model mice could be rescued by autophagy [18]. Furthermore, autophagy in mesenchymal stem cells can be influenced by different factors, such as LPS, mechanical stress, and tuberous sclerosis 1 [19–21].

Here, the effects of iRoot BP Plus on the proliferation ability and osteo/odontogenic capacity of BMMSCs were explored; the involvement of MAPK pathways and autophagy was also evaluated. Our findings revealed that iRoot BP Plus could enhance the osteogenic and odontogenic potential of BMMSCs through the MAPK pathways and autophagy.

## Materials and methods

### BMMSCs isolation

BMMSCs were collected by a whole marrow method and cultured as previously described [22]. Four-week-old Sprague-Dawley rats were purchased from the Experimental Animal Center of Nanjing Medical University and euthanized by overdose of pentobarbital, then sterilized in 75% ethyl alcohol. Then, the femora and tibiae were removed aseptically, and the bone marrow was flushed out to complete culture medium, which consists of alpha minimum essential medium (α-MEM; Gibco, Life Technologies, Grand Island, NY, USA), 10% fetal bovine serum (FBS; Hyclone, Logan, UT, USA), 100 mg/ml streptomycin, and 100 U/ml penicillin. The cells were cultured at 37 °C with 5% CO_2_. When they reached 80% confluence, BMMSCs were trypsinized and passaged. BMMSCs at passage 3–5 were used in this study.

### Material preparation

As previously reported [5], after being mixed with sterile water, ProRoot MTA (Dentsply, Tulsa, OK, USA) and iRoot BP Plus (Innovative Bioceramix, Vancouver, BC, Canada) were stored in a 100% humidified atmosphere at 37 °C for 3 days. After solidification, both materials were dried for 1 day, grounded into powder, and dissolved in α-MEM at a concentration of 20 mg/ml. The solution was then incubated at 37 °C for 3 days to obtain the bioactive ingredients of both materials. Afterwards, the supernatant was filtered and different concentrations of iRoot BP Plus-conditioned media (BP-CM) and 2 mg/ml MTA-conditioned media (MTA-CM) were prepared by mixing with fresh complete culture medium.

### Alkaline phosphatase (ALP) activity

According to the manufacturers’ protocols, total protein content was evaluated by a BCA kit (Beyotime, China) and ALP activity of each group was investigated by an ALP quantification kit (Jiancheng, Nanjing, China). ALP activity was normalized to the total protein content.

### Alkaline phosphatase staining

Following the protocol of the ALP staining kit (Beyotime, China), BMMSCs were fixed with 4% PFA, washed with PBS, and incubated in ALP working solution under a dark condition. The results were pictured and observed under a microscope.

### Alizarin Red S staining and cetylpyridinium chloride (CPC) assay

Following previous studies, BMMSCs were cultured in osteogenic-induced medium for 14 days. Osteogenic-induced medium (OM) comprised complete culture medium plus 50 mg/L ascorbic acid (Sigma-Aldrich), 10 mmol/L β-glycerophosphate (Sigma-Aldrich), and 10 nmol/L dexamethasone (Sigma-Aldrich). The medium was replaced every 3 days. At day 14, cells were fixed with 4% PFA and incubated with Alizarin Red S (Sigma-Aldrich). After washed with deionized water, cells were photographed and observed using a microscope. Ten percent CPC (Sigma-Aldrich) was used for quantification, and the spectrophotometric absorbance was examined at 560 nm. The calcium level of each group was normalized to the total protein content.

### Cell counting kit-8 assay

Cell proliferation ability was examined by cell counting kit-8 (CCK-8, Dojindo, Tokyo, Japan). BMMSCs were treated with complete culture medium or BP-CM, which was refreshed every 2 days. At days 0, 1, 3, 5, and 7, CCK-8 reagent was applied and BMMSCs were incubated at 37 °C for 1 h. The absorbance was detected at 450 nm.

### Flow cytometry (FCM)

BMMSCs cultured in different medium for 3 days were collected and fixed with 75% ethyl alcohol at 4 °C for 24 h. Afterwards, cells were washed and cell cycle fractions (G0G1/S/G2M phases) were analyzed by FACSCalibur (BD Biosciences, CA, USA).

### Real-time RT-PCR

Total cellular RNA was extracted using TRIzol reagent (Invitrogen, NY, USA). cDNA was synthesized using a PrimeScript RT Master Mix kit (TaKaRa Biotechnology, China). Primer sequences are shown in Table 1. *Gapdh* was chosen as a housekeeping gene and the relative gene expression (*Osx*, *Alp*, *Runx2*, *Opn*, and *Dspp*) was calculated using the “∆∆Ct” method.

**Table 1:** Primer sequences of real-time RT-PCR

Genes	Primers	Sequences (5′–3′)
*Osx*	Forward	GGAGGCACAAAGAAGCCATA
	Reverse	GGGAAAGGGTGGGTAGTCAT
*Alp*	Forward	GGAACGGATCTCGGGGTACA
	Reverse	ATGAGTTGGTAAGGCAGGGT
*Runx2*	Forward	TTAACGTCAGCAGGAGCAG
	Reverse	CTTCACCCCCAGGACCAAG
*Opn*	Forward	ATCTGCCGACGTACCCTTTC
	Reverse	TCGTGGCTCTGATGTTCCAG
*Dspp*	Forward	ATCTGCCGACGTACCCTTTC
	Reverse	CCTCCTGCGTGTATCCCATC
*Gapdh*	Forward	CACTGAGCATCTCCCTCACAA
	Reverse	GTATTCGAGAGAAGGGAGGGCT

*Osx* osterix, *Alp* alkaline phosphatase, *Runx2* Runt-related transcription factor 2, *Opn* osteopontin, *Dspp* dentin sialophosphoprotein, *Gapdh* D-gluteraldehyde-3-phosphate dehydrogenase

### Western blot

BMMSCs were collected to evaluate the osteo/odontogenic differentiation after BP-CM treatment at days 0, 3, and 7. BMMSCs induced with BP-CM for 0, 15, 30, and 60 min were collected to investigate the involvement of the MAPK pathways and autophagy. Cell lysates were harvested by RIPA buffer (Beyotime, China) containing 1 mM phenylmethylsulfonyl fluoride (PMSF, Beyotime, China) and 1 mM phosphatase inhibitor (Beyotime, China) on ice. Protein samples were subjected to 10% or 15% sodium dodecyl sulfate-polyacrylamide gels and transferred to PVDF membranes (Millipore, USA). After being blocked in 5% bovine serum albumin, the membranes were incubated with polyclonal antibodies overnight [OSX (ab22552, Abcam, UK), RUNX2 (ab76956, Abcam, UK), OPN (ab8448, Abcam, UK), DSPP (BS70836; Bioworld, USA), JNK (#9252, Cell Signaling Technology, USA), p-JNK (#9255, Cell Signaling Technology, USA), p38 (#8690, Cell Signaling Technology, USA), p-p38 (#4511, Cell Signaling Technology, USA), ERK (#4695, Cell Signaling Technology, USA), p-ERK (#4370, Cell Signaling Technology, USA), LC3 (#12741; Cell Signaling Technology, USA), Beclin1 (11306-1-AP; Proteintech, Chicago, USA), p62 (18420-1-AP; Proteintech, Chicago, USA), and GAPDH (#2118, Cell Signaling Technology, USA)]. Membranes were washed with PBST (PBS with 0.05% Tween 20) and immersed in secondary antibodies for 1 h. Finally, the visualization of the membranes was conducted by a Western Blot Imaging System (GE Healthcare). ImageJ software was used for quantification.

### Immunofluorescence staining

BMMSCs were fixed with 4% PAF, permeabilized with Triton X-100 solution (Beyotime, China), and blocked with goat serum. Then, cells were incubated with the primary antibodies against OSX, RUNX2, p-JNK, p-p38, p-ERK, and LC3 overnight, then incubated with a secondary antibody for 2 h under a dark condition. Nuclei were stained with DAPI (Beyotime, China). Afterwards, OSX, RUNX2, p-JNK, p-p38, and p-ERK were observed using the Olympus inverted fluorescence microscope, while LC3 was observed by the LSM 710 laser scanning confocal microscope. ImageJ software was used for quantification.

### Signal blocking assays

SP600125 (specific JNK inhibitor), SB203580 (specific p38 inhibitor), and U0126 (specific ERK inhibitor) were purchased from Selleck Chemicals (USA). According to the manufacturer’s suggestion, BMMSCs were respectively pretreated with a 10-μM inhibitor for 2 h before being stimulated with BP-CM. Cells were divided into four groups: complete culture medium, BP-CM, BP-CM+inhibitor, and complete culture medium+inhibitor. After stimulation with BP-CM for 15 min, BMMSCs were collected to evaluate the inhibition of the MAPK pathways. Western blot and immunofluorescence staining assay were performed to detect the protein levels of phosphorylation of JNK, p38, and ERK. The expression of ALP at day 5 was investigated by ALP staining and ALP activity assay. After cultured for 7 days, western blot and real-time RT-PCR were performed to evaluate the expression of osteo/odontogenic-related markers. At day 14, mineralization of BMMSCs was investigated by Alizarin Red S staining.

### Suppression of autophagy

To confirm the influence of iRoot BP Plus on autophagy in BMMSCs, 3-methyladenine (3-MA) (Sigma-Aldrich) was used to inhibit cell autophagy. As previously described [22–24], BMMSCs were respectively pretreated with 5 mM 3-MA for 24 h before being stimulated with BP-CM. Cells were divided into four groups: complete culture medium, BP-CM, BP-CM+3-MA, and complete culture medium+3-MA. After stimulation with BP-CM for 30 min, BMMSCs were collected to evaluate the suppression of autophagy. To explore the effects of autophagy on osteo/odontoblastic differentiation of BMMSCs, cells were divided into three groups: complete culture medium, BP-CM, and BP-CM+3-MA. After being cultured for 3 days, western blot and real-time RT-PCR were performed to evaluate the protein and gene levels of osteo/odontogenic-associated markers. At day 5, the expression of ALP was investigated by ALP staining and ALP activity assay. At day 14, mineralization of BMMSCs was investigated by Alizarin Red S staining.

### Transmission electron microscopy (TEM)

TEM was performed to observe autophagosomes as described previously [25]. BMMSCs were harvested and fixed with 2.5% glutaraldehyde. Then, samples were postfixed with 1% osmium tetroxide, dehydrated, and embedded. Ultrathin sections (60–80 nm) were obtained by ultramicrotome, stained with uranyl acetate and lead citrate, and examined by a transmission electron microscope (JEM-2000EX; JEOL, Ltd., Tokyo, Japan).

### Statistical analysis

Data from three independent experiments was expressed as mean ± standard deviation and analyzed by SPSS 20.0. Student’s *t* test or one-way ANOVA was used to analyze significant difference. *P* < 0.05 was considered statistical significance.

## Results

### Screening for the optimal concentration of iRoot BP Plus

**Fig. 1:**
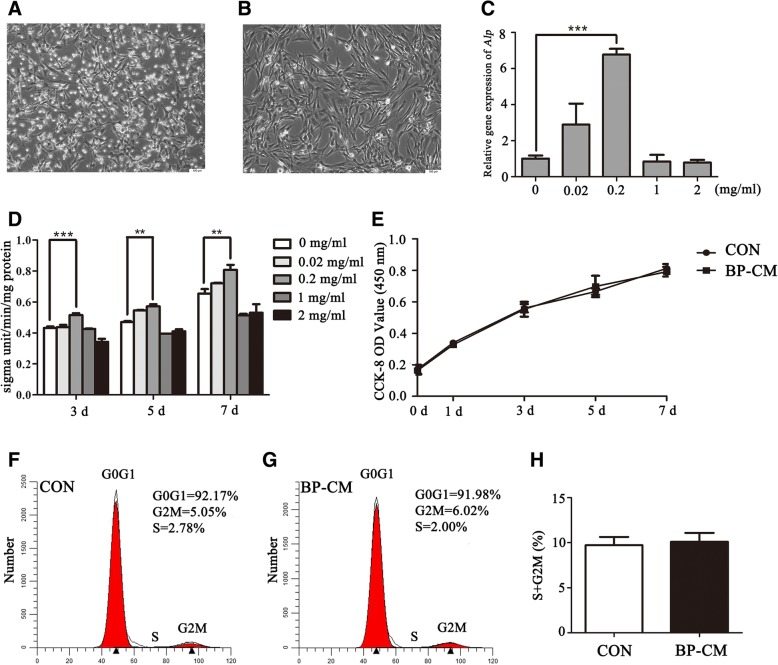
Screening for the optimal concentration of BP-CM and investigating proliferation ability of BP-CM-treated BMMSCs. **a** Primary BMMSCs at day 3. **b** BMMSCs at passage 3. Scale bar = 100 μm. **c** The gene expression of *Alp* was dramatically increased in the 0.2 mg/ml BP-CM group at day 5. ***P < 0.001. **d** ALP activity of BMMSCs cultured in different concentrations of BP-CM at days 3, 5, and 7. **P < 0.01, ***P < 0.001. **e**–**h** The proliferation ability of BP-CM-treated BMMSCs was measured by CCK-8 and FCM

Primary BMMSCs cultured after 3 days were observed as shown in Fig. 1a. The adherent cells had different cell morphologies. The round-shaped cells attached loosely and could be washed out by changing the culture medium, while the main cell type of BMMSCs at passage 3 is spindle-forming cells (Fig. 1b). At day 5, real-time RT-PCR showed that the gene expression of *Alp* was highest in the 0.2 mg/ml BP-CM group (Fig. 1c; *P* < 0.001). As compared with other groups, the 0.2 mg/ml BP-CM group also presented the highest ALP activity after being induced for 3, 5, and 7 days (Fig. 1d; *P* < 0.01). Therefore, 0.2 mg/ml was selected to be the optimal concentration for the following experiments.

### Proliferation of BP-CM-treated BMMSCs

CCK-8 assay revealed that 0.2 mg/ml BP-CM did not affect cell growth (Fig. 1e; *P* > 0.05). Flow cytometry assay showed no statistical difference in the proliferative index (PI = G2M + S) between the two groups (Fig. 1f–h; *P* > 0.05).

### iRoot BP Plus induced the osteo/odontogenic capability of BMMSCs

**Fig. 2:**
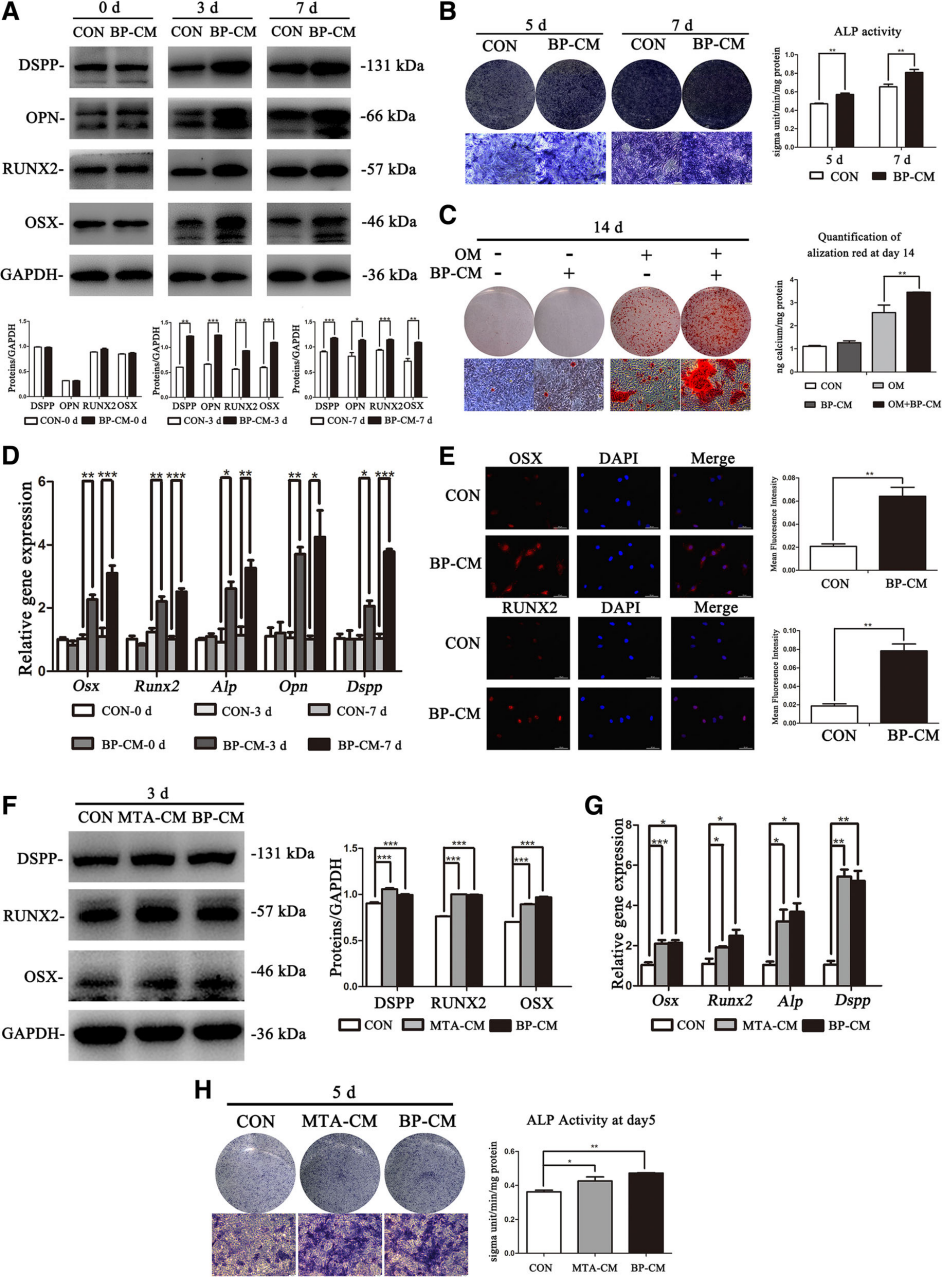
The influence of iRoot BP Plus on osteo/odontogenic differentiation of BMMSCs. **a** The osteo/odontogenic-associated proteins (DSPP, OPN, RUNX2, and OSX) in BMMSCs were detected by western blot at days 0, 3, and 7, respectively. ImageJ software was used to quantify the results, *P < 0.05, **P < 0.01, and ***P < 0.001. **b** ALP staining and ALP activity assay were performed to evaluate the expression of ALP in BP-CM-induced BMMSCs at day 5 and day 7. Scale bar = 100 μm. **P < 0.01. **c** Alizarin Red S staining and CPC assay were conducted to investigate mineralization of BMMSCs after being cultured in complete culture medium or osteogenic-induced medium (OM) (+/− BP-CM) for 14 days. Scale bar = 100 μm. **P < 0.01. **d** Relative gene expression was detected by real-time RT-PCR. *P < 0.05, **P < 0.01, and ***P < 0.001. **e** OSX and RUNX2 in BP-CM-treated BMMSCs were observed by immunofluorescence staining. Scale bar = 50 μm. Quantification was done by ImageJ, **P < 0.01. **f**, **g** Western blot and real-time RT-PCR were used to compare the changes of osteo/odontogenic-related markers in the BP-CM group with those in the MTA-CM group. *P < 0.05, **P < 0.01, and ***P < 0.001. **h** The protein level of ALP in both the BP-CM- and MTA-CM-treated BMMSCs was detected by ALP staining and ALP activity assay. Scale bar = 100 μm, *P < 0.05, **P < 0.01

The results of western blot indicated that BP-CM promoted the protein levels of dentin sialophosphoprotein (DSPP), osteopontin (OPN), runt-related transcription factor 2 (RUNX2), and osterix (OSX) in BMMSCs (Fig. 2a; *P* < 0.05). Relative gene (*Dspp*, *Opn*, *Runx2*, *Alp*, and *Osx*) expression was consistent with the proteins’ trend (Fig. 2d; *P* < 0.05). ALP was detected to examine osteogenic differentiation of BMMSCs as an early-phase marker [26]. Compared with the control group, the expression of ALP of the BP-CM group was upregulated (Fig. 2b, *P* < 0.01). Meanwhile, Alizarin Red S staining demonstrated that matrix mineralization was enhanced in the OM+BP-CM group compared with that in the OM group after induction for 14 days (Fig. 2c, *P* < 0.01). Besides, the images of immunofluorescence staining showed that the protein levels of OSX and RUNX2 were upregulated in the BP-CM group (Fig. 2e, *P* < 0.01). Above all, these data indicated that iRoot BP Plus triggered osteo/odontogenic differentiation of BMMSCs.

### The effects of iRoot BP Plus on the osteo/odontoblastic differentiation of BMMSCs were similar to those of MTA

According to our previous studies, 2 mg/ml was the optimal concentration of the MTA-conditioned medium (MTA-CM) to accelerate the osteo/odontogenic differentiation process of different stem cells [3–5]. Therefore, 2 mg/ml MTA-CM was selected to be the positive control, while a complete culture medium was used as a blank control. After being cultured for 3 days, cells were collected to extract total cellular protein and RNA. Western blot and real-time RT-PCR results revealed that both materials could upregulate the expression of osteo/odontoblastic-associated markers, but there was no significant difference between the two groups (Fig. 2f–g, *P* < 0.05). Besides, at day 5, the ALP expression was measured by ALP staining and ALP activity assay. Both BP-CM- and MTA-CM-treated BMMSCs showed higher ALP expression than the control group, and no statistical significance was observed between the two groups (Fig. 2h, *P* < 0.05). According to these data, iRoot BP Plus had a similar osteo/odontogenic capability of BMMSCs to that of MTA.

### iRoot BP Plus enhanced osteo/odontogenic potential of BMMSCs by activating the MAPK pathways

**Fig. 3:**
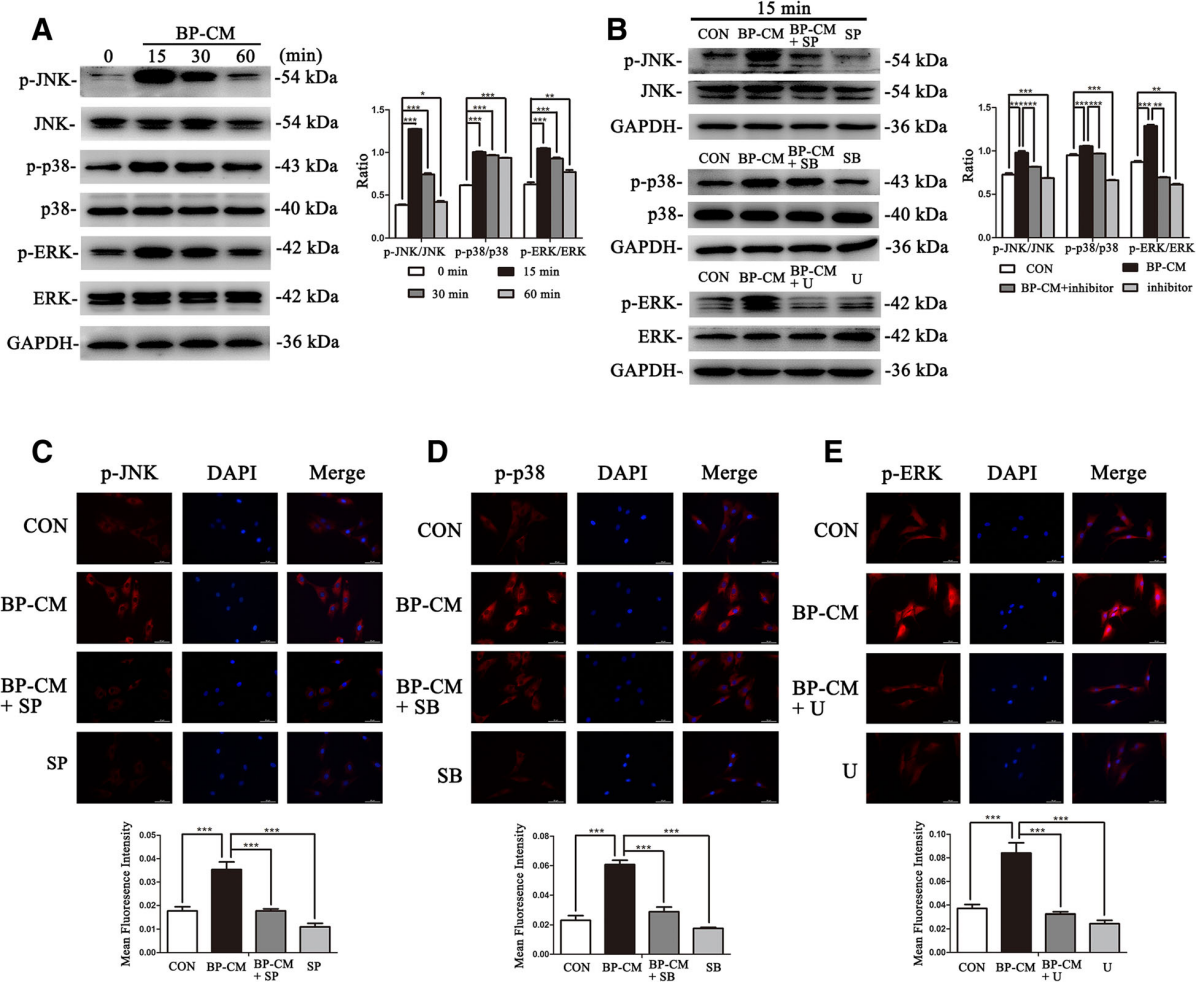
iRoot BP Plus activates the MAPK pathways in BMMSCs**.** BMMSCs of each group were cultured for an hour simultaneously, and culture medium was replaced with BP-CM at different time points (15 min, 30 min, and 60 min). **a** MAPK pathway-related proteins were investigated by western blot. Quantification was done by ImageJ. *P < 0 05, **P < 0 01, and ***P < 0 001. **b** BMMSCs were pretreated with the MAPK pathway inhibitors (SP: SP600125, SB: SB203580, and U: U0126) for 2 h and then treated with BP-CM for 15 min. Western blot was performed to investigate the MAPK pathway-related proteins. Quantitative analysis was conducted by ImageJ. **P < 0 01 and ***P < 0 001. **c**–**e** Immunofluorescence staining indicated that p-JNK, p-ERK, and p-p38 were decreased after inhibition of the MAPK pathways. Scale bar = 50 μm. Quantification was done by ImageJ, ***P < 0.001

To explore the involvement of the MAPK pathways during the BP-CM-induced differentiation of BMMSCs, the MAPK pathway-related proteins, such as p-p38, p38, p-ERK, ERK, p-JNK, and JNK, were evaluated by western blot. The results revealed that p-JNK, p-p38, and p-ERK in BP-CM-treated BMMSCs increased rapidly after 15 min and declined subsequently (Fig. 3a, *P* < 0.01). To further investigate the influence of iRoot BP Plus on the MAPK pathways in BMMSCs, inhibitors of the JNK, p38, and ERK pathways (SP: SP60016, SB: SB203580, and U: U0126) were used in this study. After being pretreated with inhibitors for 2 h, BMMSCs were treated with the complete culture medium, BP-CM, BP-CM+inhibitor, and inhibitor for 15 min and the total protein of each group was extracted. Western blot results revealed that the MAPK signaling kinase, which was activated by BP-CM, was blocked after being pretreated with specific inhibitors (Fig. 3b, *P* < 0.01). The result of immunofluorescence staining were consistent with that of western blot (Fig. 3c–e, *P* < 0.001). Therefore, MAPK pathways in BMMSCs could be instantly activated by iRoot BP Plus treatment.

### Blocking off MAPK pathways inhibited osteo/odontoblastic differentiation of iRoot BP Plus-treated BMMSCs

**Fig. 4:**
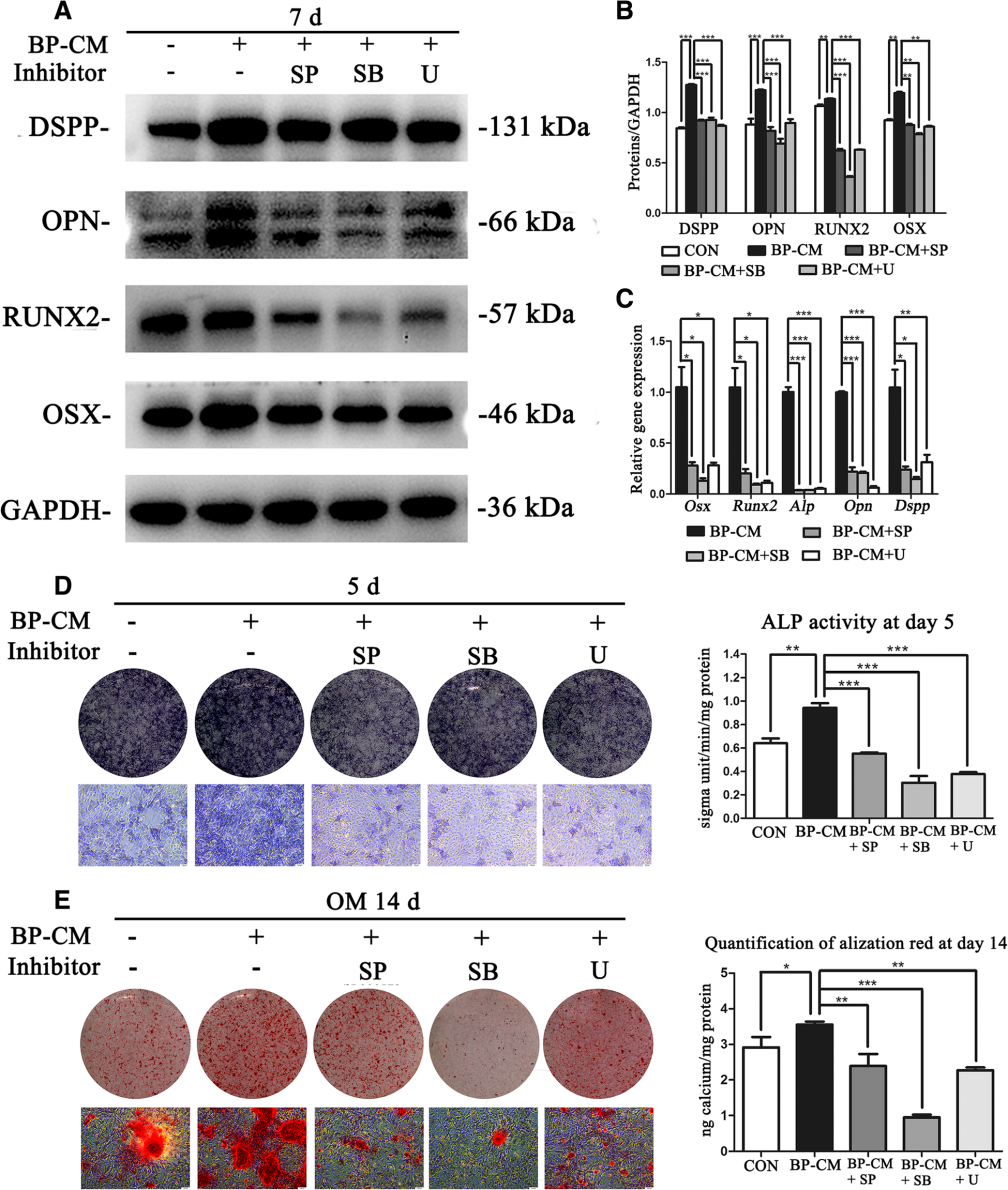
The effects of specific MAPK pathway inhibitors on osteo/odontoblastic differentiation of BP-CM-induced BMMSCs**. a**, **b** Osteo/odontogenic-related proteins were measured by western blot. Quantification was done by ImageJ, **P < 0.01 and ***P < 0.001. **c** Relative gene expression was detected by real-time RT-PCR. *P < 0.05, **P < 0.01, and ***P < 0.001. **d** ALP staining and ALP activity assay indicated that the expression of ALP in the BP-CM+inhibitor (SP600125, SB203580, and U0126) groups were downregulated as compared with those in the BP-CM group. **P < 0.01 and ***P < 0.001. **e** Alizarin Red S staining and CPC assay showed that the mineralization of BMMSCs in the BP-CM+inhibitor groups was deceased in comparison with that in the BP-CM group. Scale bar = 100 μm, **P < 0.01 and ***P < 0.001

BMMSCs were divided into five groups: complete culture medium, BP-CM, and BP-CM with different inhibitors (SP600125, SB203580, and U0126). The expression levels of odontogenic and osteogenic associated makers in BP-CM+inhibitor groups were significantly lower than those in the BP-CM group (Fig. 4a–c, *P* < 0.05). Similarly, the ALP expression in BP-CM+inhibitor groups were appreciably decreased in comparison with those in the BP-CM group (Fig. 4d, *P* < 0.01). In addition, the calcium nodules in the BP-CM+inhibitor groups, which were observed by Alizarin Red S staining, were also remarkably less and smaller than those in the BP-CM group (Fig. 4e, *P* < 0.05). Above all, these results confirmed that iRoot BP Plus enhanced osteoblastic and odontoblastic differentiation of BMMSCs through the MAPK pathways.

### iRoot BP Plus accelerate the osteo/odontogenic differentiation process of BMMSCs via autophagy

**Fig. 5:**
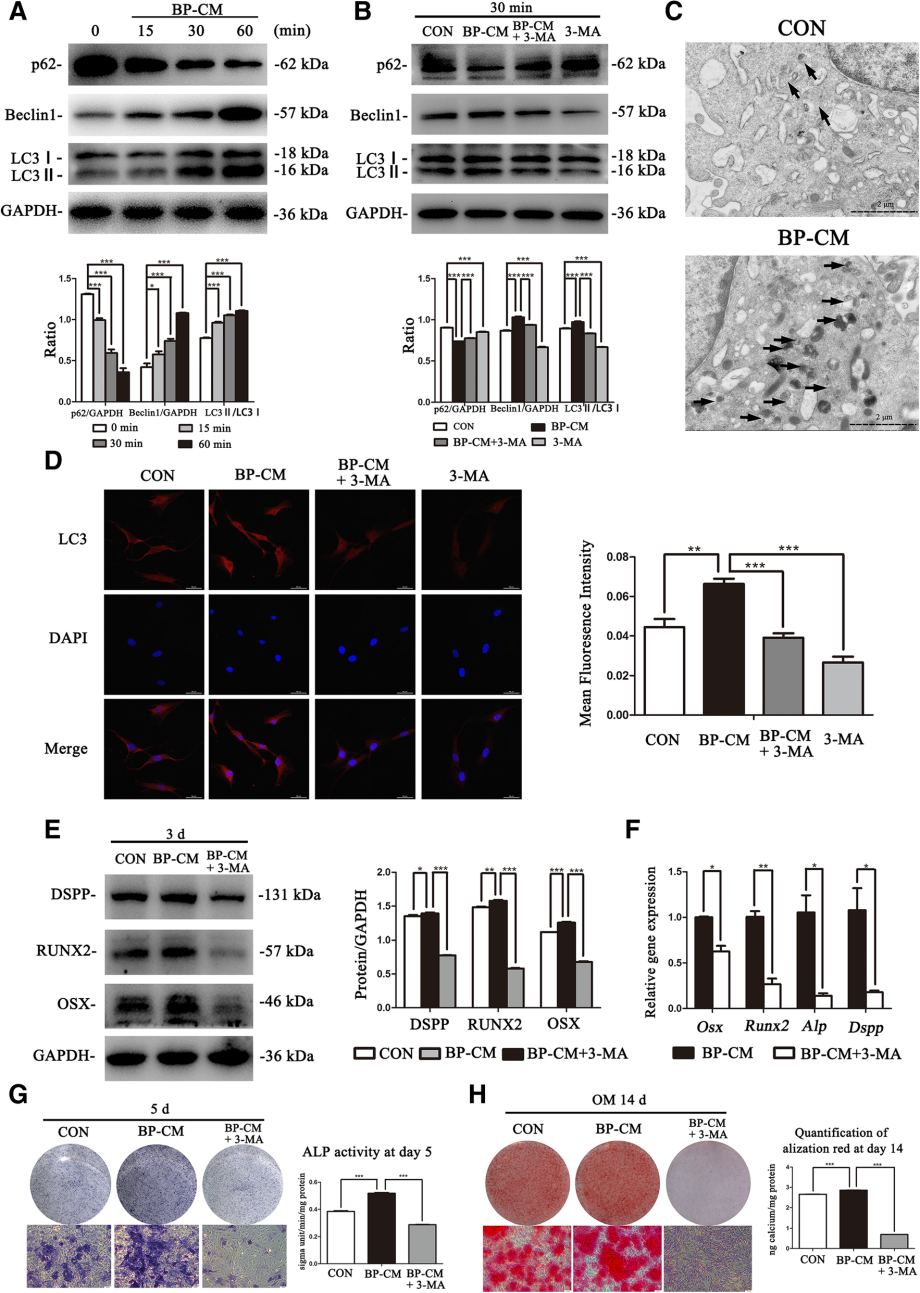
BP-CM could enhance the osteo/odontogenic capability of BMMSCs through autophagy**. a** BMMSCs of each group were cultured for an hour simultaneously, and culture medium was replaced with BP-CM at different time points (15 min, 30 min, and 60 min). Western blot was conducted to detect the autophagy-related markers (LC3, Beclin1, and p62). Quantification was done by ImageJ, *P < 0.05 and ***P < 0.001. **b** BMMSCs were divided into four groups and treated with different medium for 30 min. The normalized expression of p62 and Beclin1, and the ratio of LC3II/LC3I represented the relative expression. ***P < 0.001. **c** TEM pictures of autophagosomes showed that BP-CM-induced BMMSCs had more autophagic vacuoles as compared with cells in the control group. Autophagosomes are indicated by black arrows. Scale bar = 2 μm. **d** The images of LC3 (red) and DAPI (blue) in BMMSCs cultured with different media for 30 min were observed by immunofluorescence staining. Scale bar = 50 μm. Quantification was done by ImageJ. ***P < 0 001. **e**, **f** The effects of autophagy inhibitor 3-MA on osteo/odontoblastic-associated markers of BP-CM-induced BMMSCs were investigated by western blot and real-time RT-PCR. *P < 0.05, **P < 0.01, and ***P < 0.001. **g**, **h** Suppression of autophagy decreased the expression of ALP and mineralization of BMMSCs, while complete culture medium was served as a blank control and BP-CM was served as a positive control. Scale bar = 100 μm, ***P < 0.001

The protein levels of autophagy-related makers (p62, Beclin1, and LC3) were evaluated to determine whether 0.2 mg/ml BP-CM induced autophagy in BMMSCs. Western blot results indicated that BP-CM led to the downregulation of p62, while the protein levels of LC3II and Becline1 were increased in a time-dependent manner (Fig. 5a, *P* < 0.05). The images of TEM revealed that more autophagosomes could be observed in BP-CM-treated BMMSCs (Fig. 5c). The data above suggested that autophagy was activated during induction of osteo/odontogenic differentiation in BP-CM-treated BMMSCs. To further confirm the effects of BP-CM on autophagy of BMMSCs, 3-MA was used to suppress BP-CM-induced autophagy. As compared with the BP-CM group, p62 was decreased in BP-CM+3-MA group, while the expression of Becline1 and the ratio of LC3II/LC3I were upregulated (Fig. 5b, *P* < 0.001). Meanwhile, the results of immunostaining staining were consistent with the proteins’ trend (Fig. 5d, *P* < 0.05). To investigate the involvement of autophagy during the BP-CM-induced differentiation of BMMSCs, cells were divided into three groups: complete culture medium, BP-CM, and BP-CM+3-MA. Western blot and real-time RT-PCR indicated that the protein and gene levels of osteo/odontogenic-related markers were downregulated in the BP-CM+3-MA group in comparison with the BP-CM group (Fig. 5e, f, *P* < 0.05). At day 5, the expression of ALP in the BP-CM+3-MA group was lower than that in the BP-CM group (Fig. 5g, *P* < 0.001). After cultured for 14 days, Alizarin Red S staining showed that BMMSCs in the BP-CM group generated more calcium nodules than the BP-CM+3-MA-treated cells (Fig. 5h, *P* < 0.001). The results above demonstrated that iRoot BP Plus promoted osteo/odontogenic capability of BMMSCs through activating autophagy.

## Discussion

Accumulated evidence has demonstrated that iRoot BP Plus, a novel bioceramic material, could enhance adhesion, migration, attachment, proliferation, and mineralization ability of dental pulp stem cells [27, 28]. There are several studies focusing on the comparison between MTA, the gold standard material, and iRoot BP Plus. Liu et al. reported that iRoot BP Plus could enhance the formation of a calcium bridge at exposed pulp sites and the induction ability was superior than that of MTA [7]. On the other hand,. Shi et al. demonstrated that iRoot BP Plus is comparable with MTA when served as pulp-capping agents in dogs. Both of them formed a complete dentine bridge without pulpal inflammation, and no statistical significance was observed between the two groups [29]. Besides, iRoot BP Plus possesses superior capacity to the formation of apatite crystals as compared with MTA in vitro [30]. Moreover, iRoot BP Plus exhibited similar results to those of MTA when used as root-end filling materials in endodontic microsurgery [31].

Furthermore, similar to MTA, iRoot BP Plus contains carbon (C), oxygen (O), sodium (Na), Silicon (Si), phosphorus (P), sulfur (S), chlorine (Cl), and calcium (Ca). The major difference between the two materials is that iRoot BP Plus contains a significant amount of tantalum (Ta) and zirconium (Zr), but aluminum (Al) is not included [30]. In addition, when exposed to PBS (pH = 7.4), abundant Ca and Si was eluted from both materials [32]. Calcium takes part in the regulation of a great deal of cellular biological behavior. For example, the osteogenic differentiation of BMMSCs could be promoted by increasing concentration of extracellular calcium [33]. On the other hand, silicon could regulate the formation and calcification of bone tissue [34]. Previous studies reported that the release of Ca and Si ions contributed to the effects of calcium silicate-based biomaterials on cell differentiation [35]. Notably, during regenerative endodontic therapy, most of the cells in the blood clot contact the covering material indirectly. Thus, many researchers selected material eluates to study the biological properties of biomaterials because they can be observed conveniently and analyzed easily. In the present study, iRoot BP Plus- and MTA-conditioned media were prepared to investigate their effects on BMMSCs.

Alkaline phosphatase (ALP) has a vital function during the formation of hydroxyapatite crystal, which is the initial phase in mineralization of osteoblasts and odontoblasts [26], and its activity is upregulated at an early stage of calcification [36]. Therefore, many studies used ALP as an early-stage marker of osteo/odontoblastic differentiation [25, 37]. In the present study, 0.2 mg/ml was the optimal concentration of BP-CM to promote differentiation of BMMSCs through detecting the ALP activity at days 3, 5, and 7, as well as the gene level of *Alp* at day 5. Meanwhile, CCK-8 assay and FCM results showed that 0.2 mg/ml BP-CM had no cytotoxicity on BMMSCs. The mineralization of BP-CM-induced BMMSCs was significantly upregulated, and the expression of osteo/odontogenic markers (OPN, RUNX2, OSX, and DSPP) were remarkably increased. These genes and proteins are vital factors in regulating osteo/odontogenic differentiation at different stages [38–41], indicating that iRoot BP Plus could trigger the osteo/odontogenic differentiation process of BMMSCs. However, further research of the influence of iRoot BP Plus in vivo should be performed.

Our previous studies found that MTA-CM at a concentration of 2 mg/ml upregulate odonto/osteogenic potential of BMMSCs [3], human periodontal ligament stem cells [4], and stem cells from the apical papilla [5]. Therefore, besides the blank control group (complete culture medium), 2 mg/ml MTA-CM was served as a positive control in this study. According to the data above, iRoot BP Plus showed similar effects on osteo/odontogenic differentiation of BMMSCs to those of MTA, which was consistent with other research.

Previous studies demonstrated that the MAPK pathways have vital functions in regulating cell odontogenic and osteogenic differentiation. To date, a number of stimulators have been reported to trigger osteogenic differentiation of MSCs through the MARK pathways. For example, osteogenic capability of BMP9 could be upregulated by Win 11 via the p38 pathways [42]. Besides, extracellular calcium is reported to be an important regulator in determining the specificity of the ERK cascade [43]. In addition, several calcium silicate-based biomaterials, such as Biodentine, MTA, and Bioaggregate, could promote osteo/odontogenic potential by activating the MAPK pathways [3, 44]. In this study, p-JNK, p-ERK, and p-p38 in BMMSCs were upregulated immediately after being treated with BP-CM. Moreover, specific inhibitors (SP600125, U0126, and SB203580) could reduce the activation of BP-CM-induced MAPK pathways, respectively, and suppress the committed differentiation of BMMSCs at the same time. These results implied that iRoot BP Plus potentiated osteo/odontogenic differentiation of BMMSCs through triggering the MAPK pathways.

Autophagy is a self-cannibalization mechanism, which enables recycling of cytosolic proteins and subcellular organelles to maintain cellular homeostasis. Recently, autophagy has been recognized to be an important factor in stem cell differentiation. Aged BMMSCs have a significantly lower autophagy level than young ones, and activation of autophagy can restore degenerative properties of aged BMMSCs [22]. Meanwhile, osteogenesis could be reduced by inhibition of autophagy, while adipogenesis is upregulated [45]. Besides, autophagy could be induced when cells differentiate to osteoblasts, and a decline of autophagy-essential genes might result in the downregulation of mineralization ability. Moreover, osteoblasts use autophagic vacuoles as vehicles to secrete apatite crystals [46]. Furthermore, previous studies have revealed that various stimulators could promote osteogenic differentiation of BMMSCs through activating autophagy, such as LPS, mechanical stress, and tuberous sclerosis 1 [19–21].

LC3 has a critical function in autophagy because it governs autophagosome biogenesis. Cytoplasm LC3I and membrane-bound form LC3II are two forms of LC3. LCII was formed by the combination of LC3I and phosphatidylethanolamine during the formation of autophagosomes [24]. Thus, researchers could monitor autophagy by detecting LC3 levels. Beclin1 is another essential protein in autophagosome formation. It controls the autophagy by regulating PI3KC3-dependent generation of PI3P and the subsequent recruitment of additional ATG proteins that orchestrate autophagosome formation [47]. p62/SQSTM1 is one of the best-characterized autophagy receptors. It is a key regulator in aggrephagy, mitophagy, and xenophagy and degraded in autophagolysosomes together with the cargo [48]. Therefore, during the activation of autophagy, Beclin1 and LC3II are upregulated accompanied with a decline of p62. On the contrary, inhibition of autophagy leads to decreased Beclin1 and LC3II and results in accumulation of p62. In this study, activation of autophagy was observed during the osteo/odontogenic differentiation in BP-CM-treated BMMSCs. And suppression of autophagy resulted in the decrease of osteo/odontogenic-related markers, as well as ALP activity and mineralization of BMMSCs. These findings are consistent with the previous studies, indicating that in addition to the MAPK pathways, autophagy was also involved in osteo/odontoblastic differentiation of BMMSCs.

To date, several studies have reported the interaction between the MAPK pathways and autophagy in different types of cells during different cell behaviors. In neural stem cells, heat stress-induced autophagy and apoptosis could be reversed via inhibition of the p38 pathway [49]. 2-Phenyloxypyrimidine derivative E5 could activate the ERK pathway to induce autophagy in hepatocellular carcinoma [50]. Plakophilin 3 in ovarian cancer tissue modulates autophagy through the JNK/ERK/mTOR pathways [51]. Besides, butyrate response factor 1 regulates inflammation of RAW264.7 cells by autophagy crosstalking with the ERK pathway [52]. High glucose suppresses the migratory capacity of keratinocytes through triggering the p38 pathway which leads to downregulation of autophagy [53]. However, the relationship between autophagy and the MAPK pathways during cell osteo/odontogenic differentiation needs further investigation.

## Conclusion

In summary, 0.2 mg/ml iRoot BP Plus-conditioned medium could trigger BMMSCs differentiating to osteo/odontoblasts through the MAPK pathways and autophagy without interference with cell proliferation. Our results provide theoretical foundation on the clinical application of iRoot BP Plus in regenerative endodontic therapy.

## Data Availability

The datasets used and analyzed during the current study are available from the corresponding author on reasonable request.
